# The Man With “Pseudo-Occlusion” Syndrome: A Case Report

**DOI:** 10.4021/gr519e

**Published:** 2013-03-09

**Authors:** Apar Bains, Seema Tayal, Nada Al-Hashimi, Adesuwa Okesanya, Vasantha Kondamudi

**Affiliations:** a Department of Family Medicine, The Brooklyn Hospital Center, 121 De Kalb Avenue, Brooklyn, New York, 11201, USA

**Keywords:** Ogilvie’s syndrome, Acute colonic pseudo obstruction, Neostigmine, Colonoscopic decompression

## Abstract

We present a case of a patient with acute colonic pseudo obstruction (Ogilvie’s Syndrome) in a 63-year-old Hispanic male with multiple co morbidities, sent from the Nursing Home for evaluation of progressive abdominal distention. Clinical examination and diagnostic workup confirmed massive colonic dilatation, without mechanical obstruction.

## Introduction

Ogilvie’s Syndrome is a rare entity in which the colon becomes massively dilated without apparent mechanical obstruction. Awareness, early recognition and diagnosis are emphasized to prevent fatal complications. Although it can resolve with conservative therapy, colonoscopic decompression can prevent bowel ischemia, perforation and peritonitis.

## Case Report

A 63-year-old Hispanic male was sent in from Nursing Home for evaluation of progressive abdominal distention; which had been worsening for the last few months. He had diffuse, mild (2-3/10), dull, intermittent and non-radiating abdominal pain; which was associated with multiple episodes of non bloody, non-bilious vomiting. He also reported loose bowel motion with on and off passage of flatus, with no history of bleeding per rectum. Patient had history of similar three or four episodes of worsening abdominal distention, where in each episode mechanical obstruction was ruled out and the patient was discharged.

The patient had past medical history of paraplegia secondary to a motor vehicle accident, six years ago; peripheral neuropathy; asthma; schizophrenia; depression, dyslipidemia and hypertension. He was status post prostate surgery with suprapubic catheter placement (2009), hernia repair and IVC filter. His current medications were Flexeril, Gabapentin, Albuterol nebulizer, Seroquel, Quetiapin, Gemfibrizol and, Metoprolol. He reported history of smoking, social alcohol use but denied use of any drugs. He was allergic to morphine and opioid-like analgesics.

On examination, the patient was afebrile, with heart rate of 95 bpm, respiratory rate 20/min saturating at 97% on room air, blood pressure of 125/80 mmHg and BMI of 33.4.

The patient’s HEENT, cardiovascular and pulmonary examinations were unremarkable. On abdominal examination, it was grossly distended; bowel sounds were hypoactive, with no signs of peritoneal inflammation. Abdomen was tympanic at all four quadrants. Digital rectal examination revealed good rectal tone, no masses were detected and immediate release of hundreds of milliliters of air and passage of some loose brown stools without obvious blood was noted. Neurological exam was significant for bilateral lower extremity weakness 0/5 and loss of pain, temperature, vibration and proprioception in the legs.

**Figure 1:**
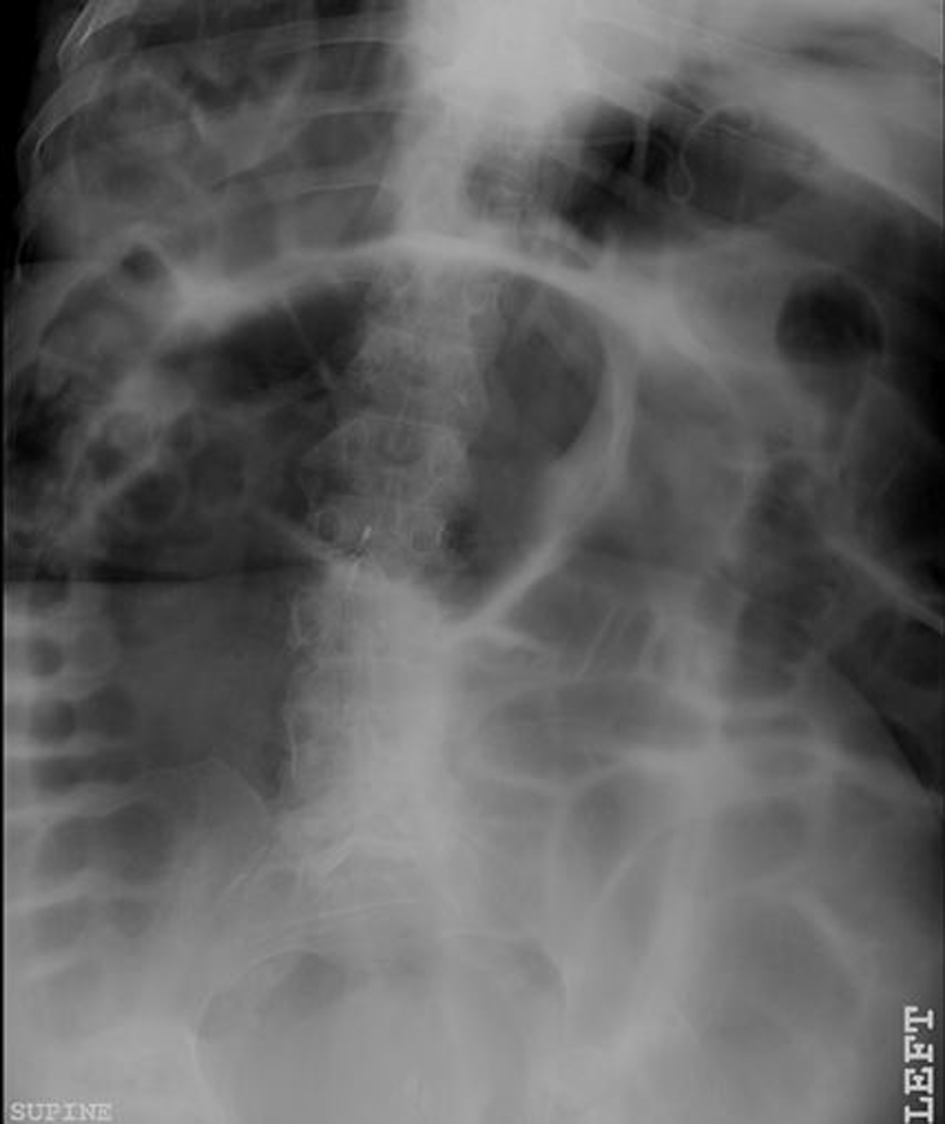
Plain X-Ray abdomen, supine position demonstrating volvulus without fluid levels.

**Figure 2:**
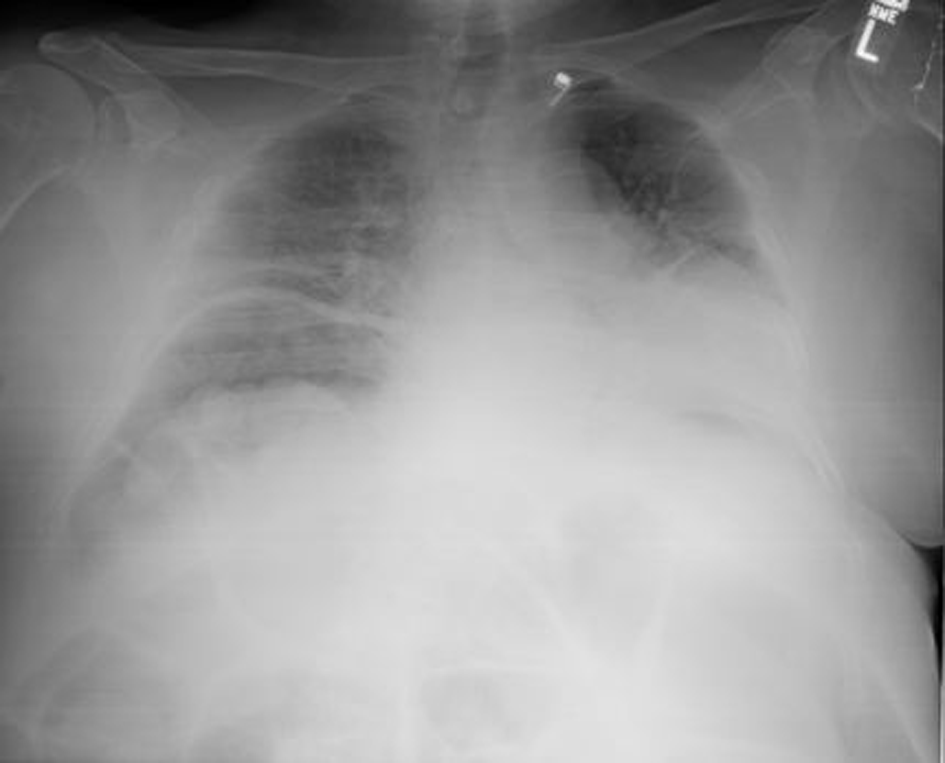
Chest X-Ray demonstrating colonic dilatation with absence of air under diaphragm.

**Figure 3:**
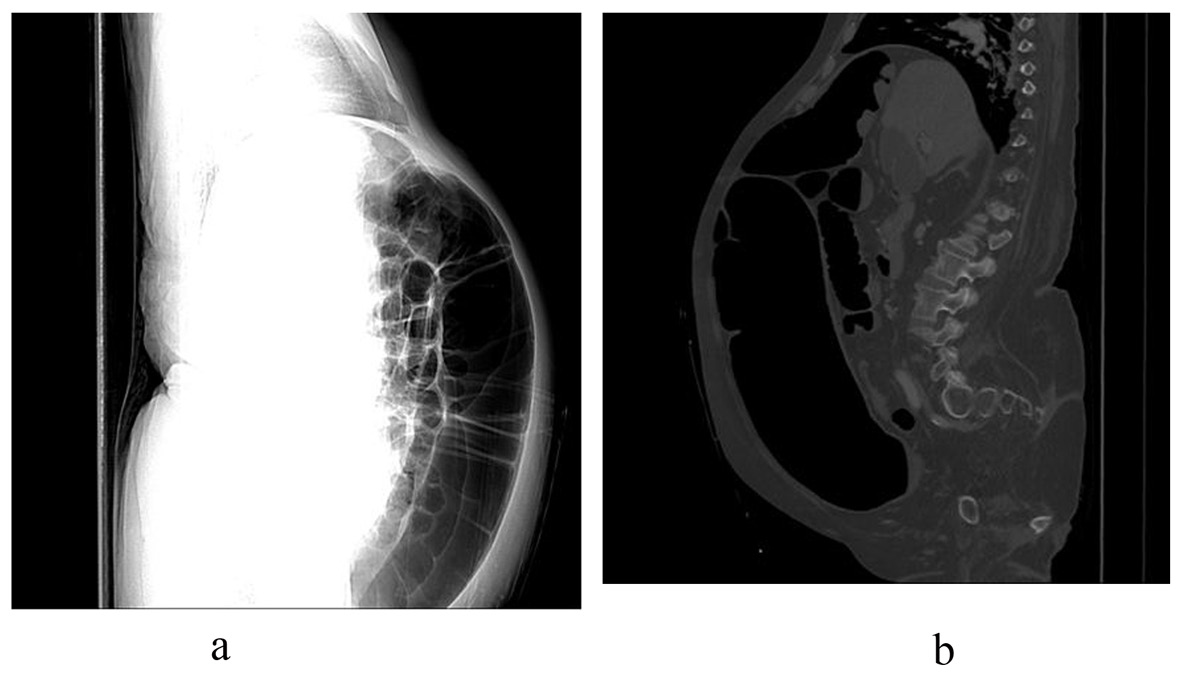
Spiral CT-Abdomen, lateral view demonstrating massive colonic dilatation.

**Figure 4:**
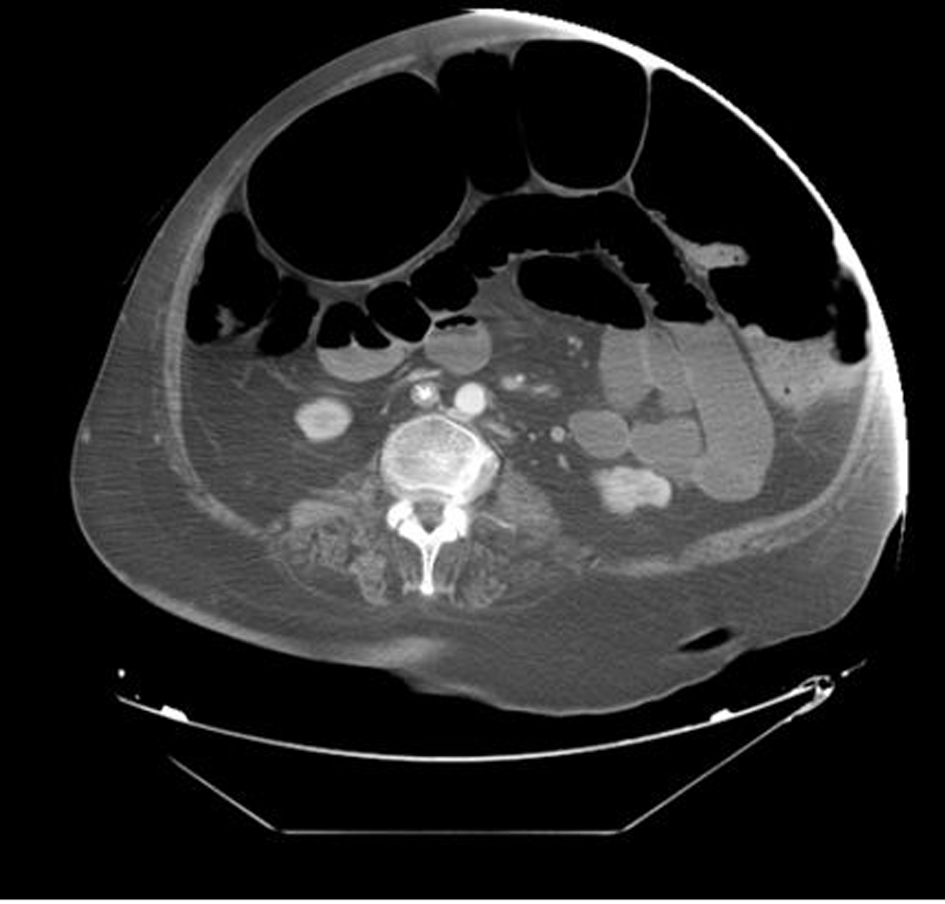
Spiral CT-Abdomen, transverse section demonstrating massive colonic dilatation.

Initial blood work-up revealed mild leukocytosis with marked hypokalemia (2.1 mmol/L). EKG showed no specific ST or T wave changes. Plain chest and abdominal X-rays showed diffusely distended bowel with volvulus without fluid levels or air under diaphragm (Fig. 1, 2). Spiral CT revealed massively distended large bowel of 5 - 6 centimeters in diameter (Fig. 3, 4). Obstruction and perforation were ruled out.

Surgery and Gastroenterology were consulted. Both consults recommended conservative management: nothing per oral, nasogastric tube, aggressive replacement of fluids and electrolytes, as well as to hold Seroquel, Flexeril and to minimize the dose of Lasix (40 mg BID to 20 mg BID). Serial abdominal X-rays were advised. Given the patient’s clinical picture, multiple co morbidities, X-ray and CT findings, his massive colonic dilatation was consistent with Acute Colonic Pseudo -Obstruction (ACPO).

On day 3, colonoscopic decompression was attempted unsuccessfully. Therefore, barium enema was done, again trial failed as well. Flexible sigmoidoscopy was then performed and it was negative. Rectal tube was placed after which distention significantly went down, volvulus resolved. Clear fluids orally were gradually introduced and tolerated. Patient ambulation was encouraged to enhance bowel motility. High fiber diet was later introduced which was tolerated as well. Patient was discharged upon his improvement on day 12 with instruction for follow up including physical activity as tolerated, close PCP follow up in the Nursing Home, as well as seeking medical attention for worsening abdominal distention.

## Discussion

Acute Colonic Pseudo-Obstruction is a motility disorder characterized by massive dilatation of the colon in the absence of an anatomic or mechanical lesion that obstructs the flow of intestinal contents. This form of adynamic ileus is also named Ogilvie’s Syndrome. It carries the name of the British surgeon Sir William Heneage Ogilvie (1887 - 1971), who first reported it, in 1948. The mean age is reported in the sixth decade, predominantly in men [1], as in our patient.

In 95% of patients it is associated with an underlying disease. The most common associations are: non-operative trauma, infection (pneumonia, sepsis), cardiac (myocardial infarction, heart failure), obstetric or gynecologic disease (Cesarean section, normal vaginal delivery), abdominal/pelvic surgery, neurological (Parkinson disease, spinal cord injury, multiple sclerosis, Alzheimer disease), orthopedic surgery, medical conditions (metabolic, cancer, respiratory failure, renal failure), and surgical conditions (urologic, thoracic, neurosurgery). There may also be an association with opiate administration and neurotransmitter blockers. Several cases are associated with spinal anesthesia used during childbirth or surgery [1].

Imbalance in autonomic sympathetic and parasympathetic neural regulation is attributed to its pathophysiology. Cecum is the usual site of dilatation, but any part of the colon can be affected. Clinically, the patient presents with massive abdominal distention, altered bowel movement with or without pain, nausea and vomiting. Presentation may be similar to paralytic ileus.

Plain abdominal roentgenogram and contrast enema are the most helpful diagnostic tools. Main complications are impending perforation of the cecum from massive colonic dilatation and colonic ischemia in diameters exceeding 12 cm, where the risk reaches 15% [2]. The awareness of the condition and its complications, early diagnosis and management can be life saving. Statistically, the mortality rate in early diagnosis is 15%, while it reaches 36-44% in delayed diagnosis [1].

Management is initially conservative and includes the following: Nothing per Oral (NPO), nasogastric tube (NGT), rectal tube, replacement of electrolyte and prone or knee chest posture if applicable [3]. Antibiotics may be given to provide some coverage for the patient when suspicion of bowel ischemia or perforation exists [4]. Choline-estrase inhibitor (Neostigmin) is being used successfully, leading to rapid colonic decompression and passage of flatus within 30 minutes [5]. Side effects of neostigmine due to parasympathetic over activity include increased salivation, increased bronchial secretions, hypotension, arrhythmia, asystole and bronchospasm. Therefore, it is recommended for the patient to be monitored in ICU. Any toxicity should be revised by Atropine. Surgery including Percutaneous endoscopic colostomy (PEC) and hemicolectomy should be limited to refractory or deteriorating patients with signs of impending ischemia or perforation. Surgical treatments have higher morbidity and mortality rates [1]. ACPO is preventable in certain conditions as in post operative hospitalized patients by early mobilization and prevention of constipation. Early recognition is important to prevent fatal complications.

## References

[1] Vanek VW, Al-Salti M (1986). Acute pseudo-obstruction of the colon (Ogilvie's syndrome). An analysis of 400 cases. Dis Colon Rectum.

[2] Kiss L, Nica C (2000). [Acute colonic pseudo-obstruction or Ogilvie syndrome]. Chirurgia (Bucur).

[3] Durai R (2009). Colonic pseudo-obstruction. Singapore Med J.

[4] Eisen GM, Baron TH, Dominitz JA, Faigel DO, Goldstein JL, Johanson JF, Mallery JS (2002). Acute colonic pseudo-obstruction. Gastrointest Endosc.

[5] Ponec RJ, Saunders MD, Kimmey MB (1999). Neostigmine for the treatment of acute colonic pseudo-obstruction. N Engl J Med.

